# Single-Cell RNA-Seq Technologies and Related Computational Data Analysis

**DOI:** 10.3389/fgene.2019.00317

**Published:** 2019-04-05

**Authors:** Geng Chen, Baitang Ning, Tieliu Shi

**Affiliations:** ^1^Center for Bioinformatics and Computational Biology, and Shanghai Key Laboratory of Regulatory Biology, Institute of Biomedical Sciences, School of Life Sciences, East China Normal University, Shanghai, China; ^2^National Center for Toxicological Research, United States Food and Drug Administration, Jefferson, AR, United States

**Keywords:** single-cell RNA-seq, cell clustering, cell trajectory, alternative splicing, allelic expression

## Abstract

Single-cell RNA sequencing (scRNA-seq) technologies allow the dissection of gene expression at single-cell resolution, which greatly revolutionizes transcriptomic studies. A number of scRNA-seq protocols have been developed, and these methods possess their unique features with distinct advantages and disadvantages. Due to technical limitations and biological factors, scRNA-seq data are noisier and more complex than bulk RNA-seq data. The high variability of scRNA-seq data raises computational challenges in data analysis. Although an increasing number of bioinformatics methods are proposed for analyzing and interpreting scRNA-seq data, novel algorithms are required to ensure the accuracy and reproducibility of results. In this review, we provide an overview of currently available single-cell isolation protocols and scRNA-seq technologies, and discuss the methods for diverse scRNA-seq data analyses including quality control, read mapping, gene expression quantification, batch effect correction, normalization, imputation, dimensionality reduction, feature selection, cell clustering, trajectory inference, differential expression calling, alternative splicing, allelic expression, and gene regulatory network reconstruction. Further, we outline the prospective development and applications of scRNA-seq technologies.

## Introduction

Bulk RNA-seq technologies have been widely used to study gene expression patterns at population level in the past decade. The advent of single-cell RNA sequencing (scRNA-seq) provides unprecedented opportunities for exploring gene expression profile at the single-cell level. Currently, scRNA-seq has become a favorable choice for studying the key biological questions of cell heterogeneity and the development of early embryos (only include a few number of cells), since bulk RNA-seq mainly reflects the averaged gene expression across thousands of cells. In recent years, scRNA-seq has been applied to various species, especially to diverse human tissues (including normal and cancer), and these studies revealed meaningful cell-to-cell gene expression variability (Jaitin et al., 2014; Grun et al., 2015; Chen et al., 2016a; Cao et al., 2017; Rosenberg et al., 2018). With the innovation of sequencing technologies, some different scRNA-seq protocols have been proposed in the past few years, which largely facilitated the understanding of dynamic gene expression at single-cell resolution (Kolodziejczyk et al., 2015; Haque et al., 2017; Picelli, 2017; Chen et al., 2018). One of them is the highly efficient strategy LCM-seq (Nichterwitz et al., 2016) which combines laser capture microscopy (LCM) and Smart-seq2 (Picelli et al., 2013) for single-cell transcriptomics without tissue dissociation. Currently available scRNA-seq protocols can be mainly split into two categories based on the captured transcript coverage: (i) full-length transcript sequencing approaches [such as Smart-seq2 (Picelli et al., 2013), MATQ-seq (Sheng et al., 2017) and SUPeR-seq (Fan X. et al., 2015)]; and (ii) 3′-end [e.g., Drop-seq (Macosko et al., 2015), Seq-Well (Gierahn et al., 2017), Chromium (Zheng et al., 2017), and DroNC-seq (Habib et al., 2017)] or 5′-end [such as STRT-seq (Islam et al., 2011, 2012)] transcript sequencing technologies. Each scRNA-seq protocol has its own benefits and drawbacks, resulting in that different scRNA-seq approaches have distinct features and disparate performances (Ziegenhain et al., 2017). In conducting single-cell transcriptomic study, specific scRNA-seq technology may need to be employed in consideration of the balance between research goal and sequencing cost.

Owing to the low amount of starting material, scRNA-seq has limitations of low capture efficiency and high dropouts (Haque et al., 2017). Compared to bulk RNA-seq, scRNA-seq produces nosier and more variable data. The technical noise and biological variation (e.g., stochastic transcription) raise substantial challenges for computational analysis of scRNA-seq data. A variety of tools have been designed to conducting diverse bulk RNA-seq data analyses, but many of those methods cannot be directly applied to scRNA-seq data (Stegle et al., 2015). Except short-read mapping, almost all data analyses (such as differential expression, cell clustering, and gene regulatory network inference) have certain disparities in methods between scRNA-seq and bulk RNA-seq. Due to the high technical noise, quality control (QC) is crucial for identifying and removing the low-quality scRNA-seq data to get robust and reproducible results. Furthermore, some analyses including alternative splicing (AS) detection, allelic expression exploration and RNA-editing identification are not suitable for the 3′ or 5′-tag sequencing protocols of scRNA-seq, but these analyses could be applicable to the data generated by whole-transcript scRNA-seq. On the other hand, an increasing number of tools are specially proposed for analyzing scRNA-seq data, and each method has its own pros and cons (Stegle et al., 2015; Bacher and Kendziorski, 2016). Therefore, to effectively handle the high variability of scRNA-seq data, attention should be paid to choosing appropriately analytical approaches.

This Review aims to summarize and discuss currently available scRNA-seq technologies and various data analysis methods. We first introduce distinct single-cell isolation protocols and various scRNA-seq technologies developed in recent years. Then we focus on the analyses of scRNA-seq data and highlight the analytical differences between bulk RNA-seq and scRNA-seq data. Considering the high technical noise and complexity of scRNA-seq data, we also provide recommendations on the selection of suitable tools to analyze scRNA-seq data and ensure the reproducibility of results.

## Isolation of Single Cells

**FIGURE 1:**
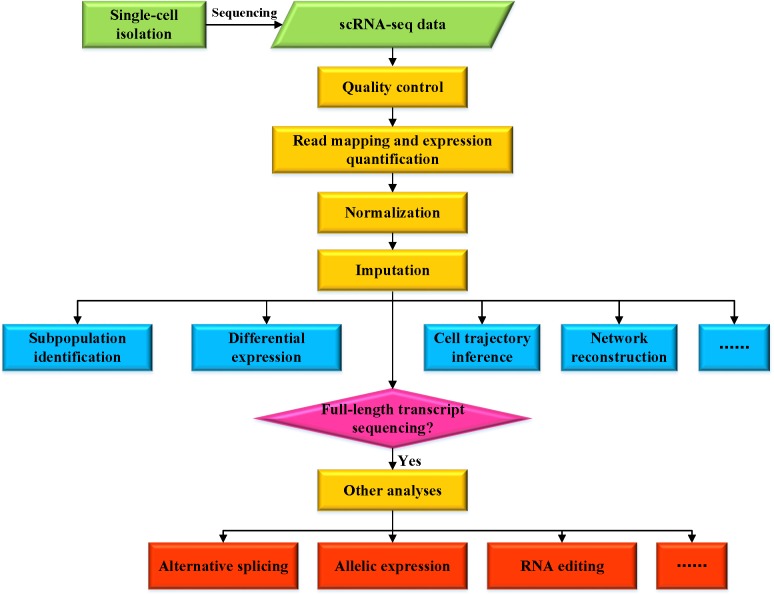
Overview of various analyses for scRNA-seq data.

The first step of scRNA-seq is isolation of individual cells (Figure 1), although the capture efficiency is a big challenge for scRNA-seq. Currently, several different approaches are available for isolating single cells, including limiting dilution, micromanipulation, flow-activated cell sorting (FACS), laser capture microdissection (LCM), and microfluidics (Gross et al., 2015; Kolodziejczyk et al., 2015; Hwang et al., 2018). Limiting dilution technique uses pipettes to isolate cells by dilution, the main limitation of this method is inefficient. Micromanipulation is a classical approach used to retrieve cells from samples with a small number of cells, such as early embryos or uncultivated microorganisms, while this technique is time-consuming and low throughput. FACS has been widely used for isolating single cells, which requires large starting volumes (>10,000 cells) in suspension. LCM is an advanced strategy used for isolating individual cells from solid tissues by using a laser system aided by computer. Microfluidics is increasingly popular due to its property of low sample consumption, precise fluid control and low analysis cost. These single-cell isolation protocols have their own advantages and show distinct performances in terms of capture efficiency and purity of the target cells (Gross et al., 2015; Hu et al., 2016).

## Currently Available ScRNA-Seq Technologies

To date, a number of scRNA-seq technologies have been proposed for single-cell transcriptomic studies (Table 1). The first scRNA-seq method was published by Tang et al. (2009), and then many other scRNA-seq approaches were subsequently developed. Those scRNA-seq technologies differ in at least one of the following aspects: (i) cell isolation; (ii) cell lysis; (iii) reverse transcription; (iv) amplification; (v) transcript coverage; (vi) strand specificity; and (vii) UMI (unique molecular identifiers, molecular tags that can be applied to detect and quantify the unique transcripts) availability. One conspicuous difference among these scRNA-seq methods is that some of them can produce full-length (or nearly full-length) transcript sequencing data (e.g., Smart-seq2, SUPeR-seq, and MATQ-seq), whereas others only capture and sequence the 3′-end [such as Drop-seq, Seq-Well and DroNC-seq, SPLiT-seq (Rosenberg et al., 2018)] or 5′-end (e.g., STRT-seq) of the transcripts (Table 1). Distinct scRNA-seq protocols may possess disparate strengths and weaknesses, and several published reviews have compared a portion of them in detail (Kolodziejczyk et al., 2015; Haque et al., 2017; Picelli, 2017; Ziegenhain et al., 2017). A previous study demonstrated that Smart-seq2 can detect a bigger number of expressed genes than other scRNA-seq technologies including CEL-seq2 (Hashimshony et al., 2016), MARS-seq (Jaitin et al., 2014), Smart-seq (Ramskold et al., 2012), and Drop-seq protocols (Ziegenhain et al., 2017). Recently, Sheng et al. (2017) showed that another full-length transcript sequencing approach MATQ-seq could outperform Smart-seq2 in detecting low-abundance genes.

**Table 1:** Summary of widely used scRNA-seq technologies.

Methods	Transcript coverage	UMI possibility	Strand specific	References
Tang method	Nearly full-length	No	No	Tang et al., 2009
Quartz-Seq	Full-length	No	No	Sasagawa et al., 2013
SUPeR-seq	Full-length	No	No	Fan X. et al., 2015
Smart-seq	Full-length	No	No	Ramskold et al., 2012
Smart-seq2	Full-length	No	No	Picelli et al., 2013
MATQ-seq	Full-length	Yes	Yes	Sheng et al., 2017
STRT-seq and STRT/C1	5′-only	Yes	Yes	Islam et al., 2011, 2012
CEL-seq	3′-only	Yes	Yes	Hashimshony et al., 2012
CEL-seq2	3′-only	Yes	Yes	Hashimshony et al., 2016
MARS-seq	3′-only	Yes	Yes	Jaitin et al., 2014
CytoSeq	3′-only	Yes	Yes	Fan H.C. et al., 2015
Drop-seq	3′-only	Yes	Yes	Macosko et al., 2015
InDrop	3′-only	Yes	Yes	Klein et al., 2015
Chromium	3′-only	Yes	Yes	Zheng et al., 2017
SPLiT-seq	3′-only	Yes	Yes	Rosenberg et al., 2018
sci-RNA-seq	3′-only	Yes	Yes	Cao et al., 2017
Seq-Well	3′-only	Yes	Yes	Gierahn et al., 2017
DroNC-seq	3′-only	Yes	Yes	Habib et al., 2017
Quartz-Seq2	3′-only	Yes	Yes	Sasagawa et al., 2018


Compared to 3′-end or 5′-end counting protocols, full-length scRNA-seq methods have incomparable advantages in isoform usage analysis, allelic expression detection, and RNA editing identification owing to their superiority of transcript coverage. Moreover, for detecting certain lowly expressed genes/transcripts, full-length scRNA-seq approaches could be better than 3′ sequencing methods (Ziegenhain et al., 2017). Notably, droplet-based technologies [e.g., Drop-seq (Macosko et al., 2015), InDrop (Klein et al., 2015), and Chromium (Zheng et al., 2017)] can generally provide a lager throughput of cells and a lower sequencing cost per cell compared to whole-transcript scRNA-seq. Thus, droplet-based protocols are suitable for generating huge amounts of cells to identify the cell subpopulations of complex tissues or tumor samples.

Strikingly, several scRNA-seq technologies can capture both polyA+ and polyA- RNAs, such as SUPeR-seq (Fan X. et al., 2015) and MATQ-seq (Sheng et al., 2017). These protocols are extremely useful for sequencing long noncoding RNAs (lncRNAs) and circular RNAs (circRNAs). Lots of studies have demonstrated that lncRNAs and circRNAs play important roles in diverse biological processes of cells and may serve as crucial biomarkers for cancers (Barrett and Salzman, 2016; Chen et al., 2016b; Quinn and Chang, 2016; Kristensen et al., 2018); therefore, such scRNA-seq methods can provide unprecedented opportunities to comprehensively explore the expression dynamics of both protein-coding and noncoding RNAs at the single-cell level.

Compared to traditional bulk RNA-seq technologies, scRNA-seq protocols suffer higher technical variations. In order to estimate the technical variances among different cells, spike-ins [such as External RNA Control Consortium (ERCC) controls (External, 2005)] and UMIs have been widely used in corresponding scRNA-seq methods. The RNA spike-ins are RNA transcripts (with known sequences and quantity) that are applied to calibrate the measurements of RNA hybridization assays, such as RNA-Seq, and UMIs can theoretically enable the estimation of absolute molecular counts. It is worth noting that ERCC and UMIs are not applicable to all scRNA-seq technologies due to the inherent protocol differences. Spike-ins are used in approaches like Smart-seq2 and SUPeR-seq but are not compatible with droplet-based methods, whereas UMIs are typically applied to 3′-end sequencing technologies [such as Drop-seq (Macosko et al., 2015), InDrop (Klein et al., 2015), and MARS-seq (Jaitin et al., 2014)]. Consequently, users can select the suitable scRNA-seq method according to the technical properties and advantages, number of cells to be sequenced and cost considerations.

## Read Alignment and Expression Quantification of ScRNA-Seq Data

The mapping ratio of reads is an important indicator of the overall quality of scRNA-seq data. Since both scRNA-seq and bulk RNA-seq technologies generally sequence transcripts into reads to generate the raw data in fastq format, no differences exist between these two types of RNA-seq data in read alignment. The mapping tools originally developed for bulk RNA-seq are also applicable to scRNA-seq data. Numerous spliced alignment programs have been designed for mapping RNA-seq data, which was extensively discussed previously (Li and Homer, 2010; Chen et al., 2011). Generally, the read mapping algorithms mainly fall into two categories: spaced-seed indexing based and Burrows-Wheeler transform (BWT) based (Li and Homer, 2010). Currently popular aligners like TopHat2 (Kim et al., 2013), STAR (Dobin and Gingeras, 2015), and HISAT (Kim et al., 2015) perform well in mapping speed and accuracy, and they can efficiently map billions of reads to the reference genome or transcriptome (Table 2). STAR is a suffix-array based method and is faster than TopHat2, but it requires a huge memory size (28 gigabytes for human genome) for read mapping (Dobin and Gingeras, 2015). Engstrom et al. systematically evaluated 26 read alignment protocols (did not include HISAT) and found that different mapping tools exhibit distinct strengths and weakness, where some programs are with a faster mapping speed but a lower accuracy in splice junction detection (Engstrom et al., 2013). HISAT is developed based on BWT and Ferragina-Manzini (FM) methods. Kim et al. (2015) showed that HISAT is currently the fastest tool that can achieve equal or better accuracy than other available aligners.

**Table 2:** Tools for read mapping and expression quantification of scRNA-seq data.

Tools	Category	URL	References
TopHat2	Read mapping	https://ccb.jhu.edu/software/tophat/index.shtml	Kim et al., 2013
STAR	Read mapping	https://github.com/alexdobin/STAR	Dobin and Gingeras, 2015
HISAT2	Read mapping	https://ccb.jhu.edu/software/hisat2/index.shtml	Kim et al., 2015
Cufflinks	Expression quantification	https://github.com/cole-trapnell-lab/cufflinks	Trapnell et al., 2010
RSEM	Expression quantification	https://github.com/deweylab/RSEM	Li and Dewey, 2011
StringTie	Expression quantification	https://github.com/gpertea/stringtie	Pertea et al., 2015


For gene/transcript expression quantification, distinct approaches are needed, based on the range of transcript sequence captured by scRNA-seq. The data generated by whole-transcript scRNA-seq methods (such as Smart-seq2 and MATQ-seq) can be analyzed with the software developed for bulk RNA-seq to quantify gene/transcript expression. Two main approaches are available for transcriptome reconstruction: *de novo* assembly (does not need a reference genome) and reference-based or genome-guided assembly (Chen et al., 2017b). *De novo* transcriptome assembly methods are primarily applied to the organisms that lack a reference genome, and are generally with a lower accuracy than that of genome-guided assembly (Garber et al., 2011). The popular genome-guided assembly tools including Cufflinks (Trapnell et al., 2010), RSEM (Li and Dewey, 2011), and Stringtie (Pertea et al., 2015) have been broadly used in many scRNA-seq studies to get relative gene/transcript expression estimation in reads or fragments per kilobase per million mapped reads (RPKM or FPKM) or transcripts per million mapped reads (TPM) (Table 2). Pertea et al. (2015) stated that StringTie outperforms other genome-guided approaches in gene/transcript reconstruction and expression quantification. On the other hand, for the 3′-end scRNA-seq protocols (e.g., CEL-seq2, MARS-seq, Drop-seq, and InDrop), specific algorithms are required to calculate gene/transcript expression based on UMIs. SAVER (single-cell analysis via expression recovery) is an efficient UMI-based tool recently proposed for accurately estimating gene expression of single cells (Huang et al., 2018). In theory, UMI-based scRNA-seq can largely reduce the technical noise, which remarkably benefits the estimation of absolute transcript counts (Islam et al., 2014).

## Quality Control of ScRNA-Seq Data

The limitations in scRNA-seq including bias of transcript coverage, low capture efficiency, and sequencing coverage result in that scRNA-seq data are with a higher level of technical noise than bulk RNA-seq data (Kolodziejczyk et al., 2015). Even for the most sensitive scRNA-seq protocol, it is a frequent phenomenon that some specific transcripts cannot be detected (termed dropout events) (Haque et al., 2017). Generally, scRNA-seq experiments can generate a portion of low-quality data from the cells that are broken or dead or mixed with multiple cells (Ilicic et al., 2016). These low-quality cells will hinder the downstream analysis and may lead to misinterpretation of the data. Accordingly, QC of scRNA-seq data is crucial to identify and remove the low-quality cells.

To exclude the low-quality cells from scRNA-seq, close attention should be paid to avoid multi-cells or dead cells in the cell capture step. After sequencing, a series of QC analyses are required to eliminate the data from low-quality cells. Those samples contain only a few number of reads should be discarded first since insufficient sequencing depth may lead to the loss of a large portion of lowly and moderately expressed genes. Then tools initially developed for QC of bulk RNA-seq data, such as FastQC^1^, can be employed to check the sequencing quality of scRNA-seq data. Moreover, after read alignment, samples with very low mapping ratio should be eliminated because they contain massively unmappable reads that might be resulted from RNA degradation. If extrinsic spike-ins (such ERCC) were used in scRNA-seq, technical noise could be estimated. The cells with an extremely high portion of reads mapped to the spike-ins indicate that they were probably broken during cell capture process and should be removed (Ilicic et al., 2016). Cytoplasmic RNAs are usually lost but mitochondrial RNAs are retained for broken cells, thus the ratio of reads mapped to mitochondrial genome is also informative for identifying low-quality cells (Bacher and Kendziorski, 2016). Additionally, the number of expressed genes/transcripts can be detected in each cell is also suggestive. If only a small number of genes can be detected in a cell, this cell is probably damaged or dead or suffered from RNA degradation. Considering the high noise of scRNA-seq data, a threshold of 1 FPKM/RPKM was usually applied to define the expressed genes. Some QC methods for scRNA-seq have been proposed (Stegle et al., 2015; Ilicic et al., 2016), including SinQC (Jiang et al., 2016) and Scater (McCarthy et al., 2017), these tools are useful for QC of scRNA-seq data.

## Batch Effect Correction

Batch effect is a common source of technical variation in high-throughput sequencing experiments. The innovation and decreasing cost of scRNA-seq enable many studies to profile the transcriptomes of a huge amount of cells. The large scale scRNA-seq data sets might be separately generated with distinct operators at different times, and could also be produced in multiple laboratories using disparate cell dissociation protocols, library preparation approaches and/or sequencing platforms. These factors would introduce systematic error and confound the technical and biological variability, leading to that the gene expression profile in one batch systematically differs from that in another (Leek et al., 2010; Hicks et al., 2018). Therefore, batch effect is a major challenge in scRNA-seq data analysis, which may mask the underlying biology and cause spurious results. To avoid incorrect data integration and interpretation, batch effects must be corrected before the downstream analysis. Because of the data feature differences between scRNA-seq and bulk RNA-seq, batch-correction approaches specially proposed for bulk RNA-seq [e.g., RUVseq (Risso et al., 2014) and svaseq (Leek, 2014)] may not be suitable for scRNA-seq. Several methods have been recently designed to mitigate the batch effects in scRNA-seq data, such as MNN (mutual nearest neighbor) (Haghverdi et al., 2018) and kBET (k-nearest neighbor batch effect test) (Buttner et al., 2019). MNN corrects the batch effects using the data from the most similar cells in different batches. KBET is a χ^2^-based method for quantifying batch effects in scRNA-seq data. These specific batch-correction approaches for scRNA-seq data can perform better than the methods developed for bulk RNA-seq (Haghverdi et al., 2018; Buttner et al., 2019).

## Normalization of ScRNA-Seq Data

To correctly interpret the results from scRNA-seq data, normalization is an essential step to get the signal of interest by adjusting unwanted biases resulted from capture efficiency, sequencing depth, dropouts, and other technical effects. Technical noise of scRNA-seq is an obvious problem due to the low starting material and challenging experimental protocols. Normalization of scRNA-seq data will benefit the downstream analyses including cell subpopulation identification and differential expression calling. In general, normalization can be divided into two different types: within-sample normalization and between-sample normalization (Vallejos et al., 2017). Within-sample normalization aims to remove the gene-specific biases (e.g., GC content and gene length), which makes gene expression comparable within one sample (such as RPKM/FPKM and TPM). In contrast, between-sample normalization is to adjust sample-specific differences (e.g., sequencing depth and capture efficiency) to enable the comparison of gene expression between samples. Generally, those simple normalization strategies are based on sequencing depth or upper quartile. If spike-ins or UMIs are used in scRNA-seq protocol, normalization can be refined based on the performance of spike-ins/UMIs (Bacher and Kendziorski, 2016).

A number of approaches have been developed for between-sample normalization of bulk RNA-seq data, such as DESeq2 (Love et al., 2014) and trimmed mean of M values (TMM) (Robinson and Oshlack, 2010). DEseq2 calculates scaling factor based on the read counts across different samples, while TMM removes the extreme log fold changes (Vallejos et al., 2017). However, bulk-based normalization approaches may be not suitable for the data of single-cell transcriptomics. Because scRNA-seq generates abundant zero-expression values and has a higher level of technical variation than bulk RNA-seq, using bulk RNA-seq normalization approaches may cause overcorrection in scRNA-seq for lowly expressed genes (Vallejos et al., 2017). Several normalization methods have been proposed for scRNA-seq data, such as SCnorm (Bacher et al., 2017), SAMstrt (Katayama et al., 2013) and a recently introduced deconvolution approach that uses the summed expression values across pools of cells to conduct normalization (Lun et al., 2016). SCnorm is based on quantile regression, while SAMstrt relies on spike-ins. Bacher et al. (2017) believed that traditional normalization methods developed for bulk RNA-seq may introduce artifacts for normalizing scRNA-seq data, while SCnorm can effectively normalize scRNA-seq data and improve principal component analysis (PCA) and the identification of differentially expressed genes.

## Imputation of ScRNA-Seq Data

Single-cell RNA sequencing data generally contain many missing values or dropouts that were caused by failed amplification of the original RNAs. The frequency of dropout events for scRNA-seq is protocol-dependent, and is closely associated with the number of sequencing reads generated for each cell (Svensson et al., 2017). The dropout events increase the cell-to-cell variability, leading to signal influence on every gene, and obscuration of gene-gene relationship detection. Therefore, dropouts can largely affect the downstream analyses since a significant portion of truly expressed transcripts may not be detectable in scRNA-seq. Imputation is a useful strategy to replace the missing data (dropouts) with substituted values. Although some methods have been proposed for imputation of bulk RNA-seq data, they are not directly applicable to scRNA-seq data (Zhang and Zhang, 2018). Several imputation methods have been recently developed for scRNA-seq, including SAVER (Huang et al., 2018), MAGIC (van Dijk et al., 2018), ScImpute (Li and Li, 2018), DrImpute (Gong et al., 2018), and AutoImpute (Talwar et al., 2018). SAVER is a Bayesian-based model designed for UMI-based scRNA-seq data to recover the true expression level of all genes. MAGIC imputes the gene expression by building Markov affinity-based graph. The developers of ScImpute suggested that SAVER and MAGIC may lead to expression changes of the genes unaffected by dropouts, while ScImpute can impute the dropout values without introducing new biases through using the information from the same genes unlikely affected by dropouts in other similar cells. DrImpute is a clustering-based approach and can effectively separate the dropout zeros from true zeros. AutoImpute is an autoencoder-based method that learns the inherent distribution of scRNA-seq data to impute the missing values. Recently, Zhang et al. evaluated different imputation methods and found that the performances of these approaches are correlated with their model hypothesis and scalability (Zhang and Zhang, 2018).

## Dimensionality Reduction and Feature Selection

Single-cell RNA sequencing data are with a high dimensionality, which may involve thousands of genes and a large number of cells. Dimensionality reduction and feature selection are two main strategies for dealing with high dimensional data (Andrews and Hemberg, 2018a). Dimensionality reduction methods generally project the data into a lower dimensional space by optimally preserving some key properties of the original data. PCA is a linear dimensional reduction algorithm, which assumes that the data is approximately normally distributed. T-distributed stochastic neighbor embedding (t-SNE) is a non-linear approach mainly designed for visualizing high dimensional data (van der Maaten and Hinton, 2008). Both PCA and t-SNE have been broadly used in diverse scRNA-seq studies to reduce the data dimension and visualize the cells discriminated into distinct subpopulations (Chen et al., 2016a; Rosenberg et al., 2018). It is worth noting that PCA cannot effectively represent the complex structure of scRNA-seq data and t-SNE has limitations of slow computation time and different embeddings for processing the same dataset multiple times. Recently, UMAP (uniform manifold approximation and projection) (Becht et al., 2018), and scvis (Ding et al., 2018) were specially developed for reducing the dimensions of scRNA-seq data. Becht et al. showed that UMAP provides the fastest run times, the highest reproducibility and the most meaningful organization of cell clusters than other dimensionality reduction approaches (Becht et al., 2018).

Feature selection removes the uninformative genes and identifies the most relevant features to reduce the number of dimensions used in downstream analysis. Reducing the number of genes by performing feature selection can largely speed up the calculations of large-scale scRNA-seq data (Andrews and Hemberg, 2018b). Differential expression is a widely used method for feature selection in bulk RNA-seq experiments, but it is hard to apply to scRNA-seq data since the information of predetermined and/or homogeneous subpopulations needed for differential expression calling of scRNA-seq data [e.g., SCDE (Kharchenko et al., 2014)] is often unavailable. Unsupervised feature selection algorithms specially designed for scRNA-seq data can be divided into the following groups: (i) highly variable genes (HVG) based; (ii) spike-in based; and (iii) dropout-based (Andrews and Hemberg, 2018a). HVG methods rely on the assumption that the genes with highly variable expression across cells are resulted from biological effects rather than technical noise. The HVG approaches include algorithms proposed by Brennecke et al. (2013), and FindVariableGenes (FVG) implemented in Seurat (Satija et al., 2015). Spike-in based approaches identify the genes showing significant higher variance than those of spike-ins with similar expression levels [e.g., scLVM (Buettner et al., 2015) and BASiCS (Vallejos et al., 2015)], which shares similar idea of HVG. Dropout based methods take advantage of the dropout distribution of scRNA-seq data to perform feature selection, like M3Drop (Andrews and Hemberg, 2018b). Andrews and Hemberg showed that their M3Drop tool outperforms existing variance-based feature selection approaches.

## Cell Subpopulation Identification

A key goal of scRNA-seq data analysis is to identify cell subpopulations (different populations are often distinct cell types) within a certain condition or tissue to unravel the heterogeneity of cells. Notably, cell subpopulation identification should be carried out after QC and normalization of scRNA-seq data, otherwise artifacts could be introduced. Approaches for clustering cells can be mainly grouped into two categories based on whether prior information is used. If a set of known markers was used in clustering, the methods are prior information based. Alternatively, unsupervised clustering methods can be used for *de novo* identification of cell populations with scRNA-seq data. The algorithms for unsupervised clustering can be primarily divided into the following types: (i) k-means; (ii) hierarchical clustering; (iii) density-based clustering; and (iv) graph-based clustering (Andrews and Hemberg, 2018a). K-means is a fast approach that assigns cells to the nearest cluster center, and it requires the predetermined number of clusters. Hierarchical clustering can determine the relationships between clusters, but it generally works slower than k-means. Density-based clustering methods need a large number of samples to accurately calculate densities and usually assume that all clusters have equal density. Graph-based clustering can be considered as an extension of density-based clustering, and it can be applied to millions of cells. Some clustering methods have been specially designed for scRNA-seq data, such as single-cell consensus clustering (SC3) (Kiselev et al., 2017) and the clustering approach implemented in Seurat (Satija et al., 2015), which can facilitate the identification of cell subpopulations (Table 3). SC3 is an unsupervised approach that combines multiple clustering approaches, which has a high accuracy and robustness in single-cell clustering. Seurat identifies the cell clusters mainly based on a shared nearest neighbor (SNN) clustering algorithm. Once the subpopulations are determined, the markers that can best discriminate distinct subpopulations are usually identified through differential expression calling or analysis of variance (ANOVA).

**Table 3:** Subpopulation identification methods for scRNA-seq data.

Methods	URL	References
SC3	http://bioconductor.org/packages/SC3	Kiselev et al., 2017
ZIFA	https://github.com/epierson9/ZIFA	Pierson and Yau, 2015
Destiny	https://github.com/theislab/destiny	Angerer et al., 2016
SNN-Cliq	http://bioinfo.uncc.edu/SNNCliq/	Xu and Su, 2015
RaceID	https://github.com/dgrun/RaceID	Grun et al., 2015
SCUBA	https://github.com/gcyuan/SCUBA	Marco et al., 2014
BackSPIN	https://github.com/linnarsson-lab/BackSPIN	Zeisel et al., 2015
PAGODA	http://hms-dbmi.github.io/scde/	Fan et al., 2016
CIDR	https://github.com/VCCRI/CIDR	Lin et al., 2017
pcaReduce	https://github.com/JustinaZ/pcaReduce	Zurauskiene and Yau, 2016
Seurat	https://github.com/satijalab/seurat	Satija et al., 2015
TSCAN	https://github.com/zji90/TSCAN	Ji and Ji, 2016


## Differential Expression Analysis of ScRNA-Seq Data

Differential expression analysis is very useful to find the significantly differentially expressed genes (DEGs) between distinct subpopulations or groups of cells. The DEGs are crucial for interpreting the biological difference between two compared conditions. The technical variability, high noise (e.g., dropouts) and massive sample size of scRNA-seq data raise challenges in differential expression calling (McDavid et al., 2013). Moreover, multiple possible cell states can exist within a population of cells, leading to the multimodality of gene expression in cells (Vallejos et al., 2016). The tools originally developed for bulk RNA-seq data have been used in many single-cell studies to identify the DEGs, but the applicability of these methods for scRNA-seq data is still unclear. In recent years, some specific methods have been proposed for conducting differential expression calling based on scRNA-seq data, such as MAST (Finak et al., 2015), SCDE (Kharchenko et al., 2014), DEsingle (Miao et al., 2018), Census (Qiu et al., 2017), and BCseq (Chen and Zheng, 2018) (Table 4). MAST is based on linear model fitting and likelihood ratio testing. SCDE is a Bayesian approach using a low-magnitude Poisson process to account for dropouts. DEsingle employs Zero-Inflated Negative Binomial model to estimate the dropouts and real zeros. BCseq mitigates the technical noise in a data-adaptive manner. Soneson and Robinson recently assessed 36 differential expression methods (including the tools designed for scRNA-seq and bulk RNA-seq data) and revealed significant differences among these approaches in the characteristics and number of DEGs (Soneson and Robinson, 2018). An increasing number of tools for differential expression analysis of scRNA-seq data will be developed, and users are encouraged to choose the tools specially designed for scRNA-seq to identify DEGs in consideration of the complex features of scRNA-seq data.

**Table 4:** Differential expression analysis tools for RNA-seq data.

Methods	Category	URL	Referenes
ROTS	Single cell	https://bioconductor.org/packages/release/bioc/html/ROTS.html	Seyednasrollah et al., 2016
MAST	Single cell	https://github.com/RGLab/MAST	Finak et al., 2015
BCseq	Single cell	https://bioconductor.org/packages/devel/bioc/html/bcSeq.html	Chen and Zheng, 2018
SCDE	Single cell	http://hms-dbmi.github.io/scde/	Kharchenko et al., 2014
DEsingle	Single cell	https://bioconductor.org/packages/DEsingle	Miao et al., 2018
Cencus	Single cell	http://cole-trapnell-lab.github.io/monocle-release/	Qiu et al., 2017
D3E	Single cell	https://github.com/hemberg-lab/D3E	Delmans and Hemberg, 2016
BPSC	Single cell	https://github.com/nghiavtr/BPSC	Vu et al., 2016
DESeq2	Bulk	https://bioconductor.org/packages/release/bioc/html/DESeq2.html	Love et al., 2014
edgeR	Bulk	https://bioconductor.org/packages/release/bioc/html/edgeR.html	Robinson et al., 2010
Limma	Bulk	http://bioconductor.org/packages/release/bioc/html/limma.html	Ritchie et al., 2015
Ballgown	Bulk	http://www.bioconductor.org/packages/release/bioc/html/ballgown.html	Frazee et al., 2015


## Cell Lineage and Pseudotime Reconstruction

The cells in many biological systems exhibit a continuous spectrum of states and involve transitions between different cellular states. Such dynamic processes within a portion of cells can be computationally modeled by reconstructing the cell trajectory and pseudotime based on scRNA-seq data. Pseudotime is an ordering of cells along the trajectory of a continuously developmental process in a system, which allows the identification of the cell types at the beginning, intermediate, and end states of the trajectory (Griffiths et al., 2018). Besides revealing the gene expression dynamics across cells, single-cell trajectory inference can also benefit the identification of the factors triggering state transitions. A number of tools have been proposed for trajectory inference, e.g., Monocle (Trapnell et al., 2014), Waterfall (Shin et al., 2015), Wishbone (Setty et al., 2016), TSCAN (Ji and Ji, 2016), Monocle2 (Qiu et al., 2017), Slingshot (Street et al., 2018), and CellRouter (Lummertz da Rocha et al., 2018) (Table 5). The resulting trajectory topology can be linear, bifurcating, or a tree/graph structure. Monocle builds a minimum spanning tree (MST) for cells to search for the longest backbone based on independent component analysis (ICA). Monocle2 uses a distinct approach that incorporates unsupervised data-driven methods with reversed graph embedding (RGE), which is more robust and much faster than Monocle. Slingshot is a cluster-based approach for identifying multiple trajectories with varying levels of supervision. CellRouter utilizes flow networks to identify cell-state transition trajectories. Recently, Saelens et al. (2018) evaluated a number of single-cell trajectory inference approaches (did not include CellRouter), and found that Slingshot, TSCAN and Monocle2 outperform other methods.

**Table 5:** Methods for single-cell trajectory inference.

Tools	Dimensionality reduction	URL	References
Monocle	ICA	http://cole-trapnell-lab.github.io/monocle-release/	Trapnell et al., 2014
Waterfall	PCA	https://www.cell.com/cms/10.1016/j.stem.2015.07.013/attachment/3e966901-034f-418a-a439-996c50292a11/mmc9.zip	Shin et al., 2015
Wishbone	Diffusion maps	https://github.com/ManuSetty/wishbone	Setty et al., 2016
GrandPrix	Gaussian Process Latent Variable Model	https://github.com/ManchesterBioinference/GrandPrix	Ahmed et al., 2019
SCUBA	t-SNE	https://github.com/gcyuan/SCUBA	Marco et al., 2014
DPT	Diffusion maps	https://media.nature.com/original/nature-assets/nmeth/journal/v13/n10/extref/nmeth.3971-S3.zip	Haghverdi et al., 2016
TSCAN	PCA	https://github.com/zji90/TSCAN	Ji and Ji, 2016
Monocle2	RGE	http://cole-trapnell-lab.github.io/monocle-release/	Qiu et al., 2017
Slingshot	Any	https://github.com/kstreet13/slingshot	Street et al., 2018
CellRouter	Any	https://github.com/edroaldo/cellrouter	Lummertz da Rocha et al., 2018


## Alternative Splicing and RNA Editing Analysis of ScRNA-Seq Data

Most of published single-cell studies mainly explored the transcriptome variation between individual cells at gene level. In eukaryotic genome, AS allows multi-exon genes to generate different isoforms, which can largely increase the diversity of both protein-coding and noncoding RNAs. Five basic modes are generally recognized for AS, including exon-skipping (cassette exon), mutually exclusive exons, alternative donor site, alternative acceptor site, and intron retention. Lots of studies have shown that AS is very common in mammalians and over 90% of human genes could undergo AS based on bulk RNA-seq data (Wang et al., 2008; Chen et al., 2017a). Moreover, AS play crucial roles in a variety of biological processes and abnormal AS may be correlated with cancers (Sveen et al., 2016). The findings revealed by bulk RNA-seq data can only reflect the averaged AS patterns of numerous cells at population level. Due to the high noise (e.g., dropouts and uneven transcript coverage) and low sequencing coverage of scRNA-seq data, the splicing quantification methods initially developed for bulk RNA-seq data are not suitable for scRNA-seq data. Since expression dynamics is a key aspect of cell populations, it is promising to study AS at single-cell resolution to gain insights into cell-level isoform usage. To date, only a few number of AS detection approaches were devised for scRNA-seq data, such as SingleSplice (Welch et al., 2016), Census (Qiu et al., 2017), BRIE (Huang and Sanguinetti, 2017), and Expedition (Song et al., 2017) (Table 6). SingleSplice uses a statistical model to detect the genes with a significant isoform usage without estimating the expression levels of full-length transcripts. Census models the isoform counts of each gene with a linear model as a Dirichlet-multinomial distribution. BRIE is a Bayesian hierarchical model for differential isoform quantification. Expedition contains a suite of algorithms for identifying AS, assigning splicing modalities and visualize modality changes. The AS detection approaches specially designed for scRNA-seq data are just emerging, thus the innovation and improvement of such methods will largely facilitate AS exploration at the single-cell level.

**Table 6:** Alternative splicing detection tools for scRNA-seq data.

Tools	URL	References
SingleSplice	https://github.com/jw156605/SingleSplice	Welch et al., 2016
Expedition	https://github.com/YeoLab/Expedition	Song et al., 2017
BRIE	https://github.com/huangyh09/brie	Huang and Sanguinetti, 2017
Census	http://cole-trapnell-lab.github.io/monocle-release/	Qiu et al., 2017


On the other hand, RNA-editing is an important post-transcriptional processing event that leads to sequence changes on RNA molecules (Gott and Emeson, 2000). Similarly, RNA-editing is mainly studied using bulk RNA-seq technologies but rarely explored at the single-cell level. Currently, the limitations of scRNA-seq largely prevented the application of RNA-editing detection to individual cells. Accordingly, with the development of both scRNA-seq technologies and single-cell editing detection algorithms, exploration of RNA-editing dynamics among single cells will be feasible. Notably, both AS and RNA-editing are mainly suitable for the data generated by scRNA-seq protocols that can sequence full-length transcripts such as Smart-seq2 and MATQ-seq rather than 3′-end scRNA-seq approaches.

## Allelic Expression Exploration with ScRNA-Seq Data

Diploid species contain two sets of chromosomes that are separately obtained from their parents. Allelic expression analysis can reveal whether genes are equally expressed between parental and maternal genomes. For autosomes, the parental and maternal expression are generally expressed equally, and aberrant expression of parental or maternal genome may cause certain diseases (McKean et al., 2016). Up to now, few methods were developed to detect the genome-wide allelic expression profile of genes based on scRNA-seq data. One main caution of allelic expression calling is that the high dropouts of scRNA-seq data may introduce many false positives. Deng et al. (2014) used a series of stringent criteria to filter the potentially false allelic calls resulted from the technical variability of scRNA-seq in studying allelic expression profile of mouse preimplantation embryos. The robustness of this strategy was further demonstrated in analyzing the dynamics of X chromosome inactivation along developmental progression using mouse embryonic stem cells (Chen et al., 2016a). SCALE was recently proposed to classify the gene expression into silent, monoallelic and biallelic, states by adopting an empirical Bayes approach (Jiang et al., 2017). We believe that allelic expression analysis at single-cell level can largely facilitate the understanding of the underlying mechanisms of dosage compensation and related diseases. It is worth noting that allelic expression investigation at single-cell level also needs the whole-transcript scRNA-seq and is mainly applicable to the organism that has available paternal and maternal single nucleotide polymorphism (SNP) information.

## Gene Regulatory Network Reconstruction

Gene regulatory network inference has been widely conducted in numerous bulk RNA-seq studies, while scRNA-seq also provides great potential for such analysis. For bulk RNA-seq data, networks are usually constructed from a number of samples using the tools like weighted gene co-expression network analysis (WGCNA) (Langfelder and Horvath, 2008; Chen et al., 2017a). A basic assumption is that the genes highly correlated in expression could be co-regulated. Because such an analysis is unable to determine the regulatory relationship, the resulting networks are typically undirected. Theoretically, the cells of scRNA-seq can be treated as the samples of bulk RNA-seq, then similar approaches are applicable to scRNA-seq data for constructing gene regulatory network.

Network inference of scRNA-seq data may reveal meaningful gene correlations and provide biologically important insights that could not be uncovered by population-level data of bulk RNA-seq. However, due to the technical noise of scRNA-seq and different subpopulations or sates of cells, attention should be paid to network reconstruction. To reduce spurious results, network inference should be carried out on each subpopulation or the cells with the same stage. Recently, Aibar et al. (2017) developed SCENIC method to reconstruct the gene regulatory network from scRNA-seq data and they showed that SCENIC can robustly predict the interactions between transcription factors and target genes. PIDC is another software designed to infer gene regulatory network from single-cell data using multivariate information theory (Chan et al., 2017). Such network inference tools facilitate the identification of expression regulatory network from single-cell transcriptomic data and provide critically biological insights into the regulatory relationships between genes.

## Conclusion

In the past 10 years, a great advancement has been achieved in scRNA-seq and a variety of scRNA-seq protocols have been developed. The development and innovation of scRNA-seq largely facilitated single-cell transcriptomic studies, leading to insightful findings in cell expression variability and dynamics. Moreover, the throughput of scRNA-seq has significantly increased with the exciting progress in cellular barcoding and microfluidics. Meanwhile, scRNA-seq methods that can be used for fixation and frozen samples have also been proposed recently, which will greatly benefit the study of highly heterogeneous clinical samples. However, currently available scRNA-seq approaches still have a high dropout problem, in which weakly expressed genes would be missed. The improvement of RNA capture efficiency and transcript coverage will definitely reduce the technical noise of scRNA-seq. Moreover, since most of current scRNA-seq methods mainly capture polyA+ RNAs, the development of protocols that can capture both polyA+ and polyA- RNAs (such as MATQ-seq) will enable comprehensive investigation of both protein-coding and non-coding gene expression dynamics at single-cell resolution.

Since the noise of scRNA-seq data is high, it is crucial to use appropriate methods to overcome the problem in analyzing scRNA-seq data. QC is necessary to exclude those low-quality cells to avoid involving artifacts in data interpretation. Furthermore, batch effect correction (if need), between sample normalization and imputation are also important and should be conducted before cell subpopulation identification, differential expression calling, and other downstream analyses. Additionally, factors such as cell size and cell cycle state could play important roles in cell variability for certain types of cells, such biases are also need to be considered. Although an increasing number of methods have been specially designed to interpret scRNA-seq data, advances of novel methods that can effectively handle the technical noise and expression variability of cells are still required. Specifically, the approaches that can accurately analyze AS and RNA-editing with scRNA-seq data are highly useful to unravel post-transcriptional mechanisms in individual cells. Overall, bioinformatics analysis of scRNA-seq data is still challenging, special attention should be paid in data interpretation, and more efficient tools are in urgent need.

Collectively, scRNA-seq and its related computational methods largely promote the development of single-cell transcriptomics. The continuous innovation of scRNA-seq technologies and concomitant advances in bioinformatics approaches will greatly facilitate biological and clinical researches, and provide deep insights into the gene expression heterogeneity and dynamics of cells.

## Author Contributions

GC and TS designed the study and wrote the manuscript. BN edited the manuscript and provided constructive comments.

## Disclaimer

The information in these materials is not a formal dissemination of the United States Food and Drug Administration.

## Conflict of Interest Statement

The authors declare that the research was conducted in the absence of any commercial or financial relationships that could be construed as a potential conflict of interest.

## Footnotes

1 https://www.bioinformatics.babraham.ac.uk/projects/fastqc/
